# Leveraging artificial intelligence in neurosurgery—unveiling ChatGPT for neurosurgical discharge summaries and operative reports

**DOI:** 10.1007/s00701-024-05908-3

**Published:** 2024-01-26

**Authors:** Daniel Dubinski, Sae-Yeon Won, Svorad Trnovec, Bedjan Behmanesh, Peter Baumgarten, Nazife Dinc, Juergen Konczalla, Alvin Chan, Joshua D. Bernstock, Thomas M. Freiman, Florian Gessler

**Affiliations:** 1 Department of Neurosurgery, University Medicine Rostock, Rostock, Germany; 2 Department of Neurosurgery, University Hospital, Schiller University Jena, Jena, Germany; 3 Department of Neurosurgery, Goethe-University Hospital, Frankfurt am Main, Germany; 4 David H. Koch Institute for Integrated Cancer Research, MIT, Cambridge, MA USA; 5 Department of Neurosurgery, Brigham and Women’s Hospital, Harvard Medical School, Boston, MA USA

**Keywords:** Artificial intelligence, AI-generated output, Computer science, Medical documentation

## Abstract

**Purpose:**

Chat generative pre-trained transformer (GPT) is a novel large pre-trained natural language processing software that can enable scientific writing amongst a litany of other features. Given this, there is a growing interest in exploring the use of ChatGPT models as a modality to facilitate/assist in the provision of clinical care.

**Methods:**

We investigated the time taken for the composition of neurosurgical discharge summaries and operative reports at a major University hospital. In so doing, we compared currently employed speech recognition software (i.e., SpeaKING) vs novel ChatGPT for three distinct neurosurgical diseases: chronic subdural hematoma, spinal decompression, and craniotomy. Furthermore, factual correctness was analyzed for the abovementioned diseases.

**Results:**

The composition of neurosurgical discharge summaries and operative reports with the assistance of ChatGPT leads to a statistically significant time reduction across all three diseases/report types: *p* < 0.001 for chronic subdural hematoma, *p* < 0.001 for decompression of spinal stenosis, and *p* < 0.001 for craniotomy and tumor resection. However, despite a high degree of factual correctness, the preparation of a surgical report for craniotomy proved to be significantly lower (*p* = 0.002).

**Conclusion:**

ChatGPT assisted in the writing of discharge summaries and operative reports as evidenced by an impressive reduction in time spent as compared to standard speech recognition software. While promising, the optimal use cases and ethics of AI-generated medical writing remain to be fully elucidated and must be further explored in future studies.

**Supplementary Information:**

The online version contains supplementary material available at 10.1007/s00701-024-05908-3.

## Introduction

Clinicians spend up to 3 h per day on medical documentation, and in many hospitals, this still involves paper charting [7]. Given advancements in deep learning and self-learning algorithms, artificial intelligence (AI) has made enormous progress in scientific writing that may ultimately be leveraged within the clinic/on the wards [14].

ChatGPT® (ChatGPT Jan 9 Version, OpenAI, USA) is a chatbot built on a powerful AI algorithm for text processing which enables it to respond to questions while concurrently adapting the style of its text output [11]. Like other models under the AI family of large language models, ChatGPT learns to understand language and generate text by predicting the next words in a passage based on the context of previous words [10]. Given that it has been trained on billions of different texts from the internet created by humans, ChatGPT can convincingly simulate scientific expertise, as recently shown by Gao et al.; this group asked ChatGPT to generate research abstracts based on distinct journal styles [5]. Resultant abstracts were exposed to an artificial intelligence (AI) output detector, plagiarism detector, and human reviewers in an effort to try and distinguish whether abstracts were original work written by humans or generated by ChatGPT [5, 13]. When given a mixture of original and generated abstracts, blinded human reviewers correctly identified 68% of generated abstracts as being generated by ChatGPT, but incorrectly identified 14% of original abstracts as being AI-generated, highlighting the power and potential of these algorithms to create realistic texts in the realm of science/medicine.

Currently, performing an objective study examining the benefits of AI in clinical practice remains difficult. Despite this, via the provision of a comparative analysis (i.e., using the current standard as a control), we have attempted to quantify measurable differences.

Using our institutional speech recognition software (SpeaKING®), we examined the time requirement(s) for neurosurgical residents to complete neurosurgical discharge summaries and operative reports and compared this with ChatGPT® for three neurosurgical conditions (i.e., chronic subdural hematoma, spinal decompression, and craniotomies).

## Methods

### Study design

Discharge notes and operative reports for patients treated between November and December 2022 at our institution were analyzed by two neurosurgical residents (4th and 5th year). Speech recognition software (SpeaKING®) was used by the residents for speech to write and then subsequently manually corrected, in all cases, and the time in minutes to complete the writing of a discharge summary and operative report (including time for manual corrections) was recorded. In total, we included 10 complication-free patients who underwent surgical management of chronic subdural hematoma, 10 complication-free one-level spinal decompression patients as well as 10 complication-free craniotomies for tumor patients in the study.

The same neurosurgical residents subsequently employed ChatGPT® to create an additional 30 discharge summaries and operative reports. The overall time was recorded in minutes for cases as per the above.

In addition, two experienced senior physicians reviewed the discharge summaries and surgical reports created by ChatGPT for factual correctness. For this purpose, a questionnaire was completed for each generated document. In the context of the selection options, the question “How factually correct do you consider this document to be?” was asked to be answered on a percentage scale of 0–100 in increments of 10.

For this study, ethical approval was obtained from the local Ethics Committee. Given that this study was a non-interventional/retrospective study, the need for patient consent was waived.

### Content(s) of the discharge summary

The minimum content requirements for the discharge summaries were medical history, neurological admission status, length of stay, type of surgery, postoperative clinical course including neuroradiological imaging, at least one laboratory-based analysis/value, neurological discharge status as well as a follow-up plan.

### Content(s) of the surgical report

The minimum content requirements for the surgical report included the patient’s history, indication for surgical treatment, documentation of the consent form, step-by-step description of the operative procedure, documentation of external material(s)/implants used, and postoperative care plan.

### Statistics

Data analyses were performed with GraphPad Software 2023 (GraphPad Software, San Diego, California, USA). For continuous parameters, the Wilcoxon/Mann–Whitney test was used. To assess the impact of the variables, odds ratios (ORs) with 95% confidence intervals (CIs) were calculated; p ≤ 0.05 was considered statistically significant.

## Results

**Fig. 1:**
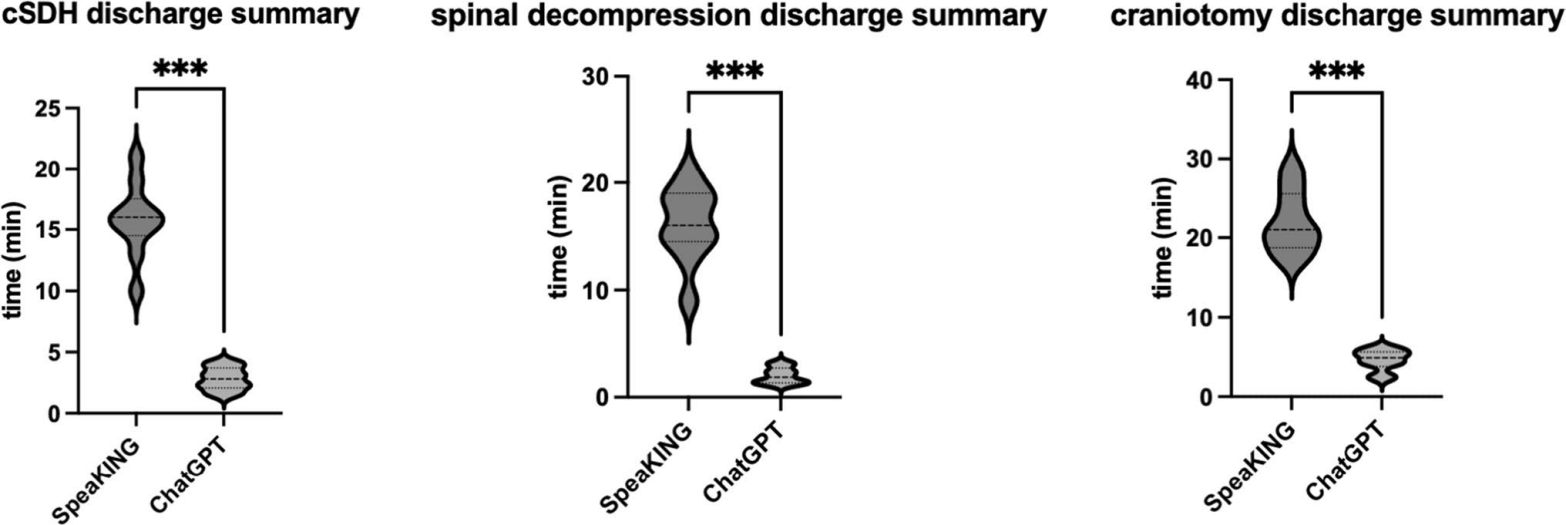
Violin plots in minutes stratified according to the discharge summary completion time with the speech recognition software SpeaKING vs ChatGPT in three distinct neurosurgical diseases. Abbreviations: cSDH, chronic subdural hematoma; ChatGPT, Chat generative pre-trained transformer. *** *p* ≤ 0.001

A total of 60 neurosurgical discharge summaries were analyzed. The median time for the generation of an inpatient discharge summary for patients who underwent surgical treatment for chronic subdural hematomas was 15 min (IQR 1.75) using SpeaKING vs 2.8 min (IQR 1.9) using ChatGPT (*p* < 0.001) (Table 1 and Fig. 1). The median time for an inpatient discharge summary for patients who underwent one-level spinal decompression was 16 min (IQR 2.85) using SpeaKING vs 2.3 min (IQR 1.3) using ChatGPT (*p* < 0.001). The median time for an inpatient discharge summary for patients who underwent craniotomies for tumor resection was 21 min (IQR 5.25) using SpeaKING vs 4.6 min (IQR 2.1) using ChatGPT (*p* < 0.001).

**Table 1:** Univariate analysis of juxtaposed time measurements according to the discharge notes completion time with speech recognition software SpeaKING vs ChatGPT in three distinct neurosurgical diseases. Abbreviations: *ChatGPT* Chat generative pre-trained transformer, *IQR* interquartile range

Discharge notes for disease type (*n* = 10)	Software		Univariate	
	ChatGPT	SpeaKING	95% CI	*p*-value
Chronic subdural hematoma, median minutes, (IQR)	1.85 (1.1)	16 (1.75)	11.72—15.84	> 0.0001
Spinal decompression, median minutes, (IQR)	1.85 (1.1)	19 (1.85)	14.31–20.25	> 0.0001
Craniotomy; median minutes, (IQR)	1.85 (1.1)	21 (5.25)	17.31—22.65	> 0.0001

**Fig. 2:**
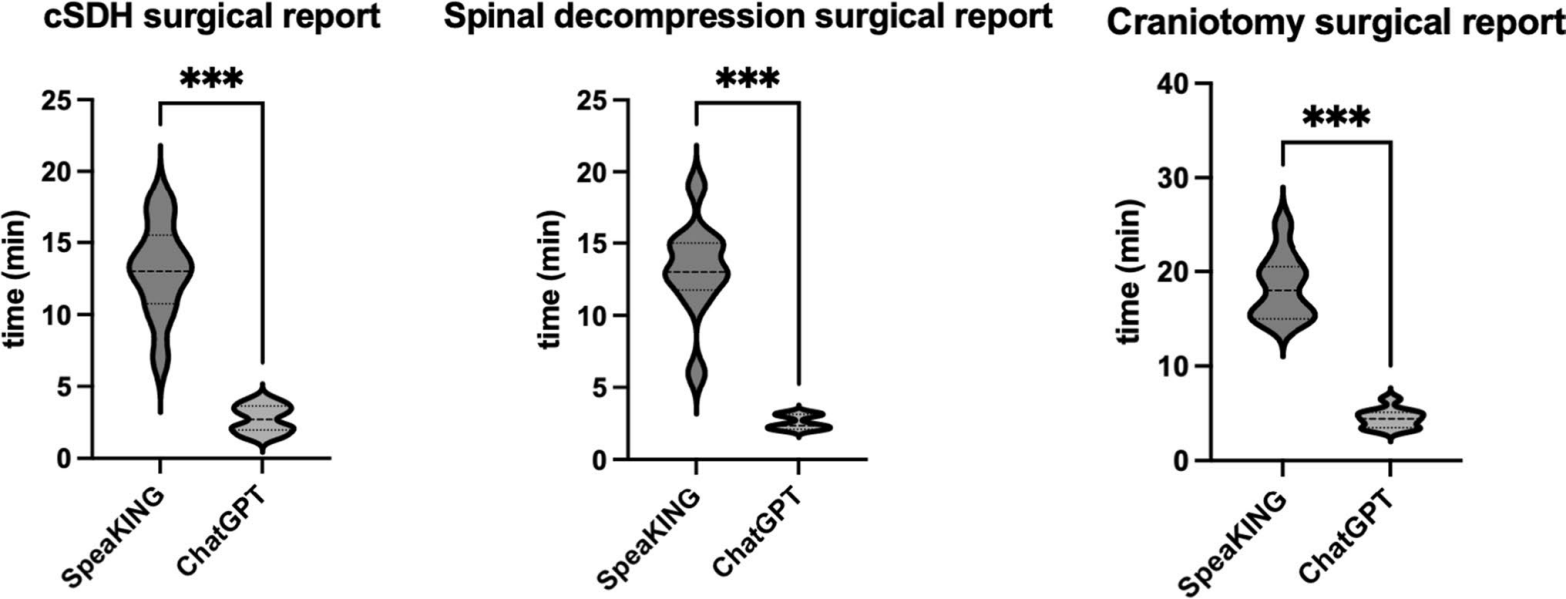
Violin plots in minutes stratified according to the operative report completion time with speech recognition software SpeaKING vs the ChatGPT in three distinct neurosurgical diseases. Abbreviations: cSDH, chronic subdural hematoma; ChatGPT, Chat generative pre-trained transformer. *** *p* ≤ 0.001

A total of 60 operative reports were also analyzed. The median time for the completion of an operative report for chronic subdural hematomas was 13.1 min (IQR 2.44) using SpeaKING vs 2.7 min (IQR 1.3) with ChatGPT (*p* < 0.001) (Table 2 and Fig. 2). The median time for the completion of an operative report for a one-level spinal decompression was 19 min (IQR 1.74) using SpeaKING vs 3.2 min (IQR 2.2) using ChatGPT (*p* < 0.001). Finally, the median time for completion of an operative report for craniotomies for tumor resection was 21 min (IQR 5.36) using SpeaKING vs 5.1 min (IQR 2.3) using ChatGPT (*p* < 0.001).

**Table 2:** Univariate analysis of juxtaposed time measurements according to the surgical report completion time with the regular speech recognition software SpeaKING vs ChatGPT in three distinct neurosurgical interventions. Abbreviations: *ChatGPT* Chat generative pre-trained transformer, *IQR* interquartile range

Surgical reports (*n* = 10)	Software		Univariate	
	ChatGPT	SpeaKING	95% CI	*p*-value
Chronic subdural hematoma, median minutes, (IQR)	2.7 (1.3)	13.1 (2.44)	8.12–12.61	< 0.001
Spinal decompression, median minutes, (IQR)	3.2 (2.2)	19 (1.74)	8.36–12.88	< 0.001
Craniotomy, median minutes, (IQR)	5.1 (2.3)	21 (5.36)	11.58–26.30	< 0.001

**Fig. 3:**
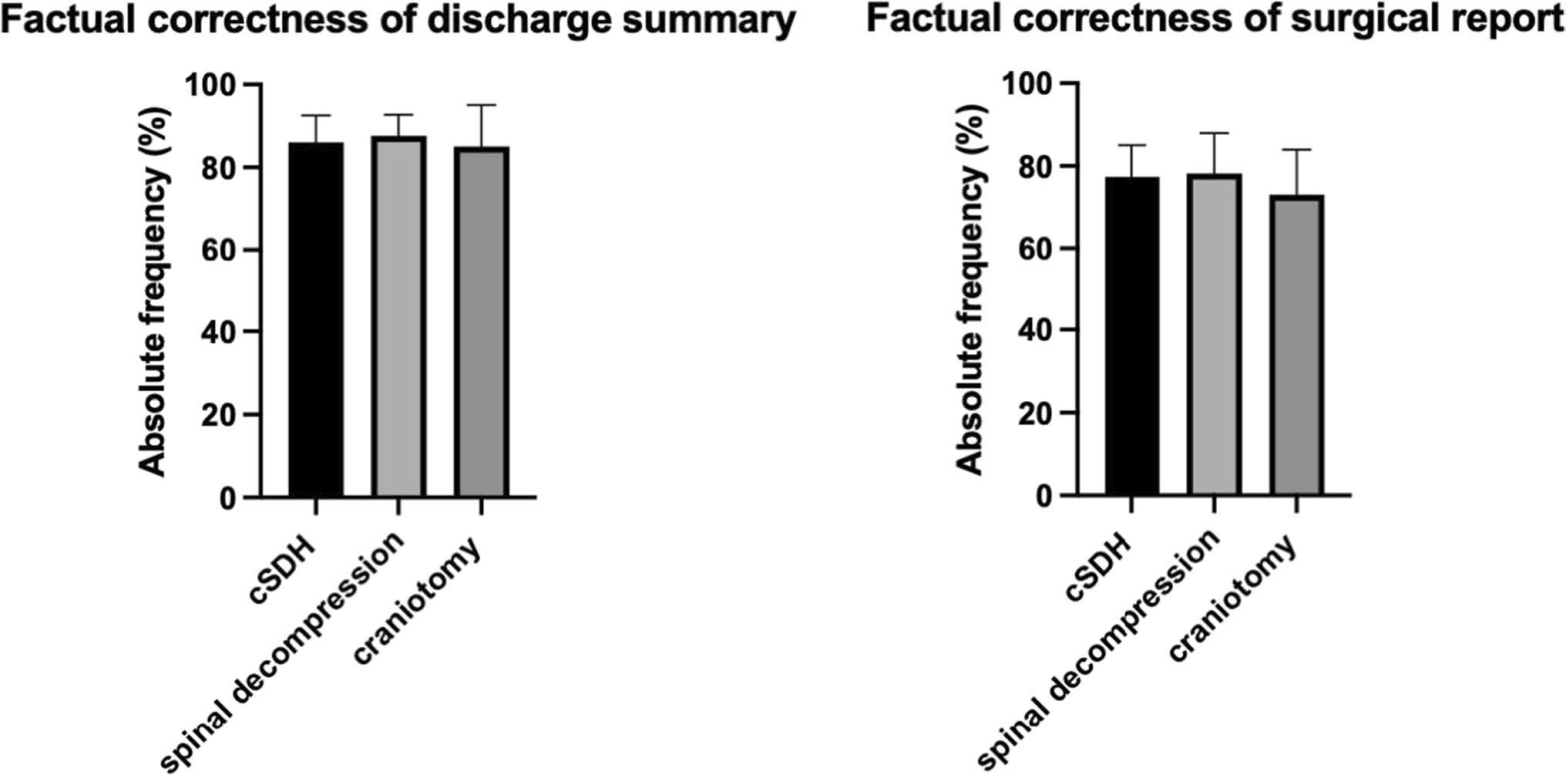
Box plots in percent stratified according to the factual correctness of discharge summaries and surgical reports as analyzed by two experienced attendings in three distinct neurosurgical diseases. Abbreviations: cSDH, chronic subdural hematoma; ChatGPT, Chat generative pre-trained transformer

A total of 30 neurosurgical discharge summaries were analyzed. The median percentage for factual correctness for cSDH was 83%, 85% for spinal decompression, and 81% for craniotomy. Furthermore, a total of 30 surgical reports showed factual correctness as 78% for cSDH, 79% for spinal decompression, and 71% for craniotomy. In a statistical analysis within the disease pattern, comparing the factual correctness of cSDH for discharge summary vs surgical report, we found no statistical significance (*p* = 0.512). Furthermore, in a statistical analysis comparing the factual correctness of spinal decompression for discharge summary vs surgical report, we found no statistical significance (*p* = 0.642). However, the comparison of discharge summary vs surgical report for craniotomy showed a significant reduction in factual correctness for craniotomy surgical reports (*p* = 0.002) (Fig. 3).

## Discussion

Our study highlights the ability of ChatGPT to assist in the completion of inpatient clinical tasks as evidenced by a reduction in time to completion as compared to speech recognition software for both neurosurgical discharge summaries and operative reports. Interestingly, ChatGPT was able to generate nearly perfect discharge summaries and operative reports via the employment of accurate medical terms and subject-specific language. (For exemplary illustration see supplementary Fig. 1.)

Given these findings, several arguments can be derived that support the implementation of ChatGPT into a clinical routine(s); in particular, deploying ChatGPT in settings centered on repetitive/chart-based work may significantly improve resident satisfaction/reduce computer time [12]. Furthermore, automated documentation, the placing of routine medical orders, and/or interactions with insurers may ultimately be automated [8, 9]. Another relevant benefit of this novel technology is the possibility for non-native speakers to engage ChatGPT as a tool and in so doing decrease the burden of writing/formatting, thereby improving the quality of resultant products and the experience of the clinical provider [4].

Given that discharge summaries constitute an essential component of the transition from inpatient to outpatient settings, the implementation/use of modern technologies that improve the quality/speed for which these reports can be completed is critical.

On the other hand, given that this technology is nascent and has not yet been sufficiently studied/applied in clinical setting, risks must also be highlighted and discussed [1]. For example, since ChatGPT is capable of generating human-like text, it has the potential to impersonate physicians online and/or spread misinformation [6]. ChatGPT and other large language models are prone to hallucination, generating factually incorrect but grammatically fluent content underscoring the need for safeguard in critical use cases [2]. Specifically, our analyses here show the danger of inadequate factual correctness for surgical reports on craniotomies.

Recently, privacy concerns about ChatGPT’s company (OpenAI) access to users’ data used to generate text have culminated in its world’s first nationwide ban in Italy [3]. As such, it is of paramount importance to minimize the potential for abuse of patient data and ensure proper access controls are in place. Accordingly, future studies are warranted in an effort to identify the optimal use(s) and ethical boundaries of AI-assisted medical writing.

For instance, it is important to recognize the practical challenges that ChatGPT faces when instructed to create surgical reports for complex neurosurgical interventions i.e., eloquent brain areas, since the ability of ChatGPT to produce precise and contextually appropriate surgical reports depends on patterns discovered from enormous amounts of data, which frequently include more common complication-free surgeries. Therefore, the collaboration between ChatGPT and the neurosurgeon is essential, given the difficulties presented by complicated and unusual cases. Particularly, complex interventions with unexpected intraoperative decisions require individualized and context-sensitive reporting, where the neurosurgeons’ knowledge and skillset are unmatched.

Furthermore, for the correct interpretation of the time-saving aspect, the reader must take the different completion times by the attendings into account, a fact that could not be investigated in this study.

Moreover, for proper ChatGPT usage in the future, it is of paramount importance to fully elucidate the complications and unforeseen moments in neurosurgical procedures in the published literature. Not only is this data necessary to deliver and improve ChatGPT-generated neurosurgical output, but a transparent complication report is also an indispensable marker for modern-day hospital.

## Limitations

Our study has several limitations which include a relatively small sample size and applications. Furthermore, as per our study protocol, confounding, selection bias, and uncontrolled statistical error risks cannot be definitely excluded. Future studies should seek to engage large cohorts and expand the possible use cases for ChatGPT in clinical care while seeking to ensure the fidelity of protected patient data.

## Conclusions

ChatGPT is a promising tool with the potential to free up clinicians, thereby allowing clinical members of the care team to spend more time on important/meaningful portions of clinical encounters. Our results preliminary highlight two potential applications in neurosurgery that may allow for improvements in patient care/management.

## Supplementary information

ESM 1 **Supplementary Figure 1.** Screenshot of a textual description of a sample surgical note for a single level spinal decompression generated by ChatGPT. (PNG 511 kb)
