# Supplementary figures and images for: Leveraging artificial intelligence in neurosurgery—unveiling ChatGPT for neurosurgical discharge summaries and operative reports
Source: Acta Neurochir (Wien). 2024 Jan 26;166(1):38. doi: 10.1007/s00701-024-05908-3 (PMC10817836; doi:10.1007/s00701-024-05908-3)

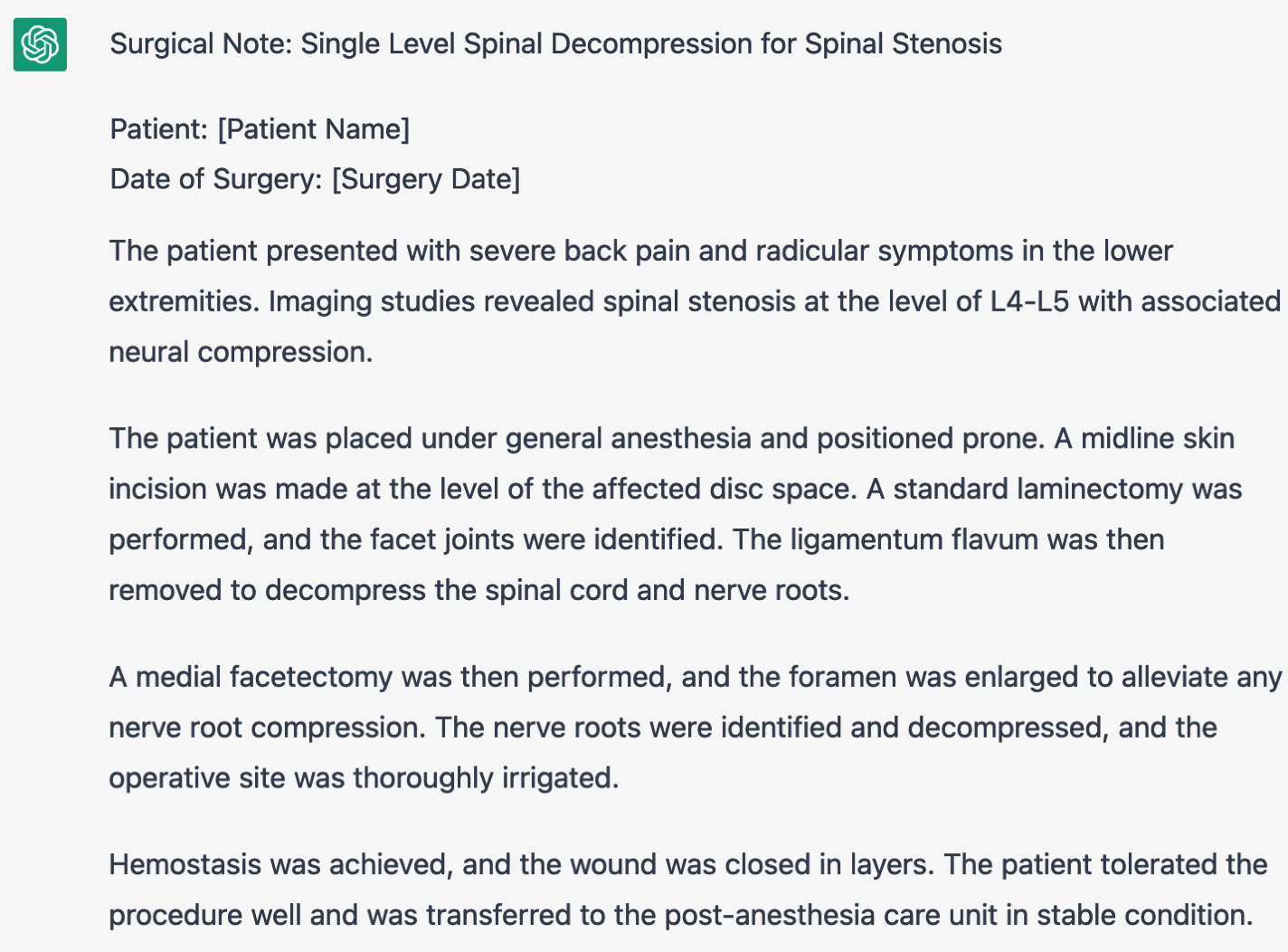

Supplement: Supplementary file 1 — Supplementary Figure 1. Screenshot of a textual description of a sample surgical note for a single level spinal decompression generated by ChatGPT. (PNG 511 kb) [file 701_2024_5908_MOESM1_ESM.png]
